# Low Perceived Self-Efficacy Impedes Discriminative Fear Learning

**DOI:** 10.3389/fpsyg.2019.01191

**Published:** 2019-06-18

**Authors:** Friederike Raeder, Lioba Karbach, Helena Struwe, Jürgen Margraf, Armin Zlomuzica

**Affiliations:** Ruhr University Bochum, Faculty of Psychology, Mental Health Research and Treatment Center, Bochum, Germany

**Keywords:** self-efficacy, negative feedback, fear conditioning, expectancy learning, affective learning

## Abstract

Perceived self-efficacy refers to a subject’s expectation about the outcomes his/her behavior will have in a challenging situation. Low self-efficacy has been implicated in the origins and maintenance of phobic behavior. Correlational studies suggest an association between perceived self-efficacy and learning. The experimental manipulation of perceived self-efficacy offers an interesting approach to examine the impact of self-efficacy beliefs on cognitive and emotional functions. Recently, a positive effect of an experimentally induced increased self-efficacy on associative learning has been demonstrated. Changes in associative learning constitute a central hallmark of pathological fear and anxiety. Such alterations in the acquisition and extinction of conditioned fear may be related to cognitive and neurobiological factors that predict a certain vulnerability to anxiety disorders. The present study builds on previous own work by investigating the effect of an experimentally induced low perceived self-efficacy on fear acquisition, extinction and extinction retrieval in a differential fear conditioning task. Our results suggest that a negative verbal feedback, which leads to a decreased self-efficacy, is associated with changes in the acquisition of conditioned fear. During fear acquisition, the negative verbal feedback group showed decreased discrimination of fear responses between the aversive and safe conditioned stimuli (CS) relative to a group receiving a neutral feedback. The effects of the negative verbal feedback on the acquisition of fear discrimination learning were indexed by an impaired ability to discriminate the probability of receiving a shock during acquisition upon presentation of the aversive (CS+) relative to the safe stimuli (CS−). However, the effects of low self-efficacy on discrimination learning were limited to fear acquisition. No differences between the groups were observed during extinction and extinction retrieval. Furthermore, analysis of other outcome measures, i.e., skin conductance responses and CS valence ratings, revealed no group differences during the different phases of fear conditioning. In conclusion, lower perceived self-efficacy alters cognitive/expectancy components of discrimination during fear learning but not evaluative components and physiological responding. The pattern of findings suggests a selective, detrimental role of low(er) self-efficacy on the subject’s ability to learn the association between ambiguous cues and threat/safety.

## Introduction

Anxiety disorders belong to the most frequent and chronic mental disorders. The associative learning model has been used as a valid experimental model for deriving a mechanistic understanding on how pathological fear and anxiety is developed and maintained in the course of different anxiety disorders (Mineka and Zinbarg, 1996, 2006; Pittig et al., 2018). Alterations in associative learning have been proposed as a central hallmark of pathological fear and anxiety (Lissek et al., 2005). In line with this idea, a great deal of evidence suggests that subjects with anxiety disorders exhibit enhanced conditionability, i.e., a faster and stronger acquisition of conditioned fear as well as a delayed and detrimental extinction of conditioned fear responses (Lissek et al., 2005; Duits et al., 2015). Moreover, systematic changes in conditionability might constitute a causal link, which predispose certain individuals to develop anxiety or stress-related disorders (Otto et al., 2007). Accordingly, longitudinal studies demonstrate that individual differences in the acquisition and extinction of conditioned fear contribute to an increased risk to develop anxiety symptoms after exposure to a traumatic event (Guthrie and Bryant, 2006; Lommen et al., 2013). Thus, the associative learning model has been increasingly acknowledged as a translational tool to identify cognitive and neurobiological difference factors that predict a certain vulnerability to pathological fear and anxiety (Mineka and Oehlberg, 2008; Lonsdorf and Merz, 2017; Pittig et al., 2018).

People can differ substantially in their belief of being able to exercise control over demanding and emotionally relevant situations. The latter has been defined as self-efficacy, a core concept of the social cognitive theory, which received considerable interest in different research domains during the last decades (Bandura, 1997; Maddux, 1999). In terms of clinical significance, changes in perceived self-efficacy have been presumed to be implicated in the origins and maintenance of phobic behavior (Williams, 1995; Bandura, 1997; Maddux, 1999). The perceived belief to cope with potentially threatening situations contributes to anxiety arousal levels (Bandura, 1988). In particular, it has been shown that subjects who display an increased belief to be capable of exercising control over potential threats tend to show decreased anxiety levels (Muris, 2002). Accordingly, low levels of perceived self-efficacy have been associated not only with higher levels of trait anxiety/neuroticism but also with more frequent symptoms of anxiety disorders (Muris, 2002). Furthermore, low self-efficacy levels are associated with greater severity of anxiety (Richards et al., 2002; Thomasson and Psouni, 2010) and an increased usage (Thomasson and Psouni, 2010). Interestingly, both heightened trait anxiety (Chan and Lovibond, 1996) and state anxiety (Dibbets and Evers, 2017) have been linked to systematic alterations in fear conditioning. While these preliminary findings suggest a mutual relationship between perceived self-efficacy, anxiety levels, and conditioning processes which could contribute to the development and maintenance of pathological fear and anxiety, the evidence so far is restricted to correlative analyses.

The experimental manipulation of perceived self-efficacy by means of a positive/negative verbal feedback offers an interesting approach to modulate the subject’s level of self-efficacy and to examine the impact of this intervention on cognitive and emotional functions. In recent years, this approach has been successfully employed, having an effect on different cognitive functions which are related to certain psychopathologies, i.e., aversive learning, episodic future thinking, and problem solving capacity (e.g., Brown et al., 2012a,b). We have recently shown that positive verbal persuasion increases self-efficacy levels which in turn promotes fear extinction learning and its retrieval (Zlomuzica et al., 2015). Our findings thus provide first evidence for a direct impact of perceived self-efficacy on associative learning in the context of Pavlovian conditioning. In particular, our data suggest that an increased self-efficacy might contribute to an enhanced regulation of conditioned fear responses. Thus, increasing perceived self-efficacy might be beneficial in demanding and threatening situations and might contribute to an increased coping capability.

The present study builds on this previous work and investigates whether a lower level of self-efficacy is associated with changes in conditionability. To this end, a detrimental effect of negative verbal persuasion on self-efficacy and aversive learning (Brown et al., 2012b) and problem solving capacity (Brown et al., 2012a) has been demonstrated. Based on these previous studies, we tested whether an experimental manipulation (i.e., a negative verbal persuasion) aimed at decreasing perceived self-efficacy is associated with concomitant changes in fear acquisition, extinction and/or extinction retrieval. Associative learning was investigated in the context of Pavlovian conditioning, i.e., during a differential fear conditioning task. Generally, conditioning processes can be quantified across different outcome measures. These include physiological indices of human fear responding, such as skin conductance responses (SCRs) and subjective measures of fear responding. The latter refers to verbal report measures, including both cognitive/expectancy (CS-US contingency ratings) and evaluative/affective (CS valence ratings) components of fear conditioning. In this instance, the US-expectancy measure in fear conditioning research has a high diagnostic validity with respect to anxiety disorders (Boddez et al., 2013). Overestimation of danger probability related to feared stimuli might be a central feature of phobia (Jones and Menzies, 2000). Interestingly, the level of self-efficacy in phobic individuals is highly related to avoidance behavior (Jones and Menzies, 2000). Thus, assessing the effect of an experimental manipulation of self-efficacy beliefs on cognitive/expectancy components of fear conditioning might be of special importance to the psychopathology of fear and anxiety (Boddez et al., 2013). Nevertheless, in the present study, we employed the simultaneous recording of different indices of fear learning to elucidate the possible effect of a self-efficacy manipulation on different processes of fear learning (see Baeyens et al., 1995; Hamm and Vaitl, 1996; Hamm and Weike, 2005).

## Materials and Methods

### Participants

*N* = 71, ranging in age from 18 to 36 years, were recruited *via* bulletin board notices at the campus of the Ruhr-Universität Bochum or *via* postings in social media networks. Participation was restricted to healthy participants who had no current mental or neurological diseases. Five participants had to be excluded from data analysis due to the presence of a mental disorder (*n* = 2) and technical software failure (*n* = 3). Hence, the analytic sample comprised 66 participants (*n* = 33 in each experimental group). All experimental procedures were approved by the local ethics committee of the Ruhr-Universität Bochum and were carried out in accordance with the Declaration of Helsinki. All participants provided written informed consent and were reimbursed with 15€ or 1.5 course credits.

### Fear Conditioning

The unconditioned stimulus (US) was a 500 ms mild electrical stimulation generated by a Constant Current Isolated Stimulator PS3 (Digitimer Ltd., Welwyn Garden City, England). The US was delivered to the skin of the lower (dominant) arm *via* Ag/AgCl electrodes.

The reinforced and non-reinforced conditioned stimuli (CS+ and CS−, respectively) were black-and-white or yellow-and-blue inkblot pictures, with the allocation of these pictures to the CS+ and CS− being counterbalanced across participants. The CS+ and CS− were presented for 8 s on a black 19-inch computer screen. The intertrial interval varied randomly between 16 and 20 s. Stimulus delivery was controlled with Presentation software.

**Figure 1:**
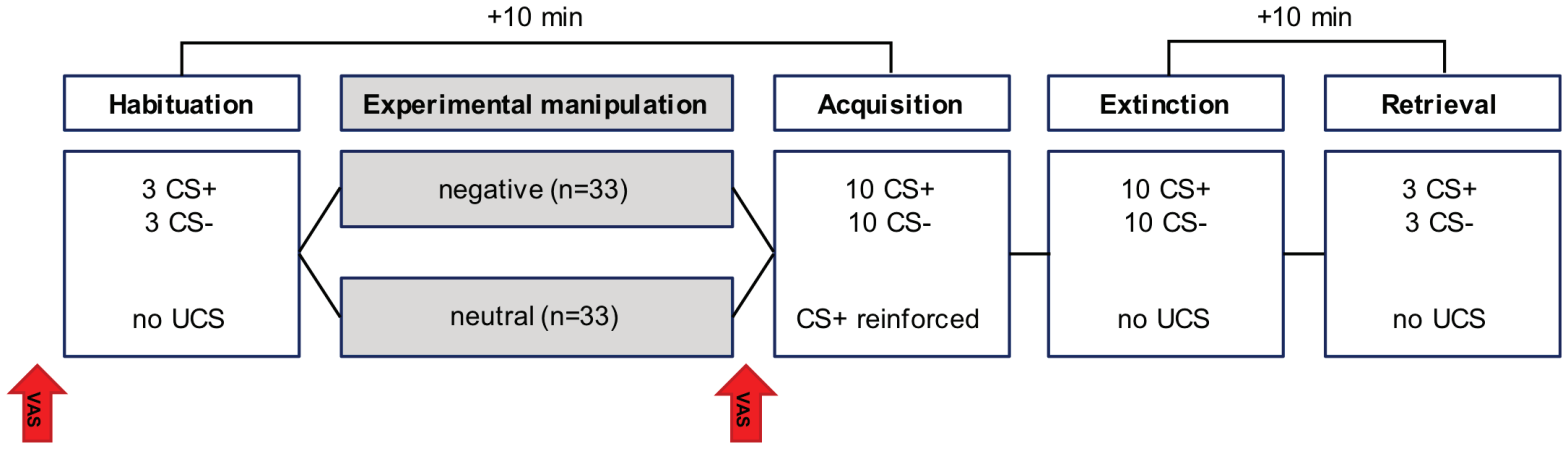
Outline of the fear conditioning paradigm and the experimental manipulation. The arrows denote the manipulation check. Breaks of 10 min were imposed between habituation and acquisition (to deliver the feedback) as well as between extinction and retrieval.

As displayed in Figure 1, fear conditioning comprised four phases: habituation (3 CS+, 3 CS−), acquisition (10 CS+, 10 CS−), extinction (10 CS+, 10 CS−), and retrieval (3 CS+, 3 CS−). The CS+ co-terminated with the US on a 70% reinforcement schedule only during the acquisition phase. The CS− was never presented with the US. Breaks of 10 min were imposed between habituation and acquisition as well as between extinction and retrieval.

### Experimental Manipulation

Participants were randomly assigned to receive either a negative or a neutral feedback which was administered after the habituation phase of fear conditioning (see Figure 1). The negative verbal feedback was slightly modified from Brown et al. (2012b). Briefly, participants were told that the responses given to the questionnaires and their psychophysiological responses recorded thus far provided a measure on how they cope in demanding situations. Based on this measure, they had been classified as being in the lower 30–50 percentile of “copers” (= negative feedback; for details, see Brown et al., 2012b) or as exhibiting no abnormalities (= neutral feedback).

### Assessments

#### Fear Conditioning

##### CS Valence and CS-US Contingency Ratings

After each conditioning phase, CS valence (“how pleasant do you feel when you see this picture”) and CS-US contingency (“do you think that this picture is paired with an electrical stimulation”) ratings were obtained with visual analogue scales displayed on the computer screen. The anchor points ranged from 0 (CS valence: very pleasant; CS-US contingency: extremely unlikely) to 100 (CS valence: very unpleasant; CS-US contingency: extremely likely).

##### SCRs

SCRs were measured *via* 5 mm inner diameter Ag/AgCl electrodes. The electrodes were filled with non-hydrating electrodermal paste. The index and middle finger of participants’ non-dominant hand were used for electrode placement. Physiological data were recorded at a sampling rate of 1000 Hz using a 16-bit Brain Amp Exg Amplifier and Brain Vision recorder software (version 1.2, Brain products GmbH, Gilching, Germany). Conditioned SCRs were obtained by subtracting the average skin conductance level for the 1000 ms prior to CS onset from the maximum SCL recorded during the 8000 ms that followed CS onset. SCRs that fell below 0.1 μS were scored as zero-responses (Dawson et al., 2007). SCR data were transformed with the natural logarithm (ln(μS + 1)) to account for skewness (Dawson et al., 2007).

#### Questionnaires

The 21-item Depression Anxiety Stress Scales (DASS-21; Lovibond and Lovibond, 1995) were used to assess depression, anxiety, and stress levels among participants. General self-efficacy beliefs were measured with the General Self-efficacy Scale (GSE; Jerusalem and Schwarzer, 1999) and emotion regulation capabilities with the Emotion Regulation Questionnaire (ERQ; Abler and Kessler, 2009). Manipulation checks were undertaken, measuring participants’ current level of distraction, excitement, positive and negative mood, as well as perceived coping capability on five visual analogue scales (VAS), which ranged from 0 (= minimum) to 100 (= maximum). These were given both before the fear conditioning task (i.e., before habituation) as well as after the experimental manipulation (i.e., before acquisition, see Figure 1).

### Procedure

Participants were informed about the course of the study. As part of the instruction, each participant was informed that the experiment involves the presentation of two pictures on the computer screen. The participants were informed that one of these stimuli may sometimes co-occur with an electric stimulation. They were further instructed that questionnaires would be filled in at pre-determined time-points and that their physiological responses would be collected continuously during the entire experiment. Both would be evaluated in parallel by the experimenter, who would derive a performance measure and provide a feedback at some point during the experimental procedure. After written informed consent was obtained, electrodes for US delivery and SCR measurements were fitted. The US was adjusted to a level participants experienced as unpleasant but not painful (according to a standardized shock work-up, comprising at most five sample shocks to individually adjust US intensity, see Heitland et al., 2013). Participants then filled in the VAS, GSE, and ERQ. Subsequently, the rating scales (CS valence and CS-US contingency) were practiced. Thereafter, the fear conditioning task commenced with the experimenter delivering the verbal feedback (negative or neutral) during the break between habituation and acquisition. After the feedback had been given, the VAS was again filled in and the acquisition phase was started. In the second break, participants completed the demographic questionnaire and the DASS. At the end of the experimental procedure, participants were fully debriefed about the feedback they had received.

### Statistical Analysis

Statistical analyses were carried out in IBM SPSS 24 for Windows. Manipulation checks were assessed with a series of mixed ANOVAs with time (pre-manipulation vs. post-manipulation) and group (negative vs. neutral) on each of the five visual analogue scales. Fear conditioning data were subjected to mixed ANOVAs, conducted separately for each conditioning phase (i.e., habituation, acquisition, extinction, and extinction retrieval) and outcome measure (i.e., CS valence, CS-US contingency, and SCRs). CS (CS+ vs. CS−) was entered as within-subjects factor and group (negative vs. neutral) as between subjects factor. In analyzing SCR data during the acquisition and extinction phases, block (early vs. late; averaged across the first and last five trials, respectively) was entered as an additional within-subjects factor. A result was considered significant at *p* < 0.05.

## Results

### Participant Characteristics

The neutral and negative groups were comparable in age (neutral: *M* = 25.8, SD = 4.2, negative: *M* = 24.7, SD = 4.0, *p* = 0.31), gender proportion (%female: neutral: 70%, negative: 55%, *p* = 0.31), scores on the DASS total (neutral: *M* = 7.97, SD = 6.02, negative: *M* = 11.03, SD = 7.05, *p* = 0.06) as well as in each subscale of the DASS (all *p* > 0.09). Furthermore, both group showed no significant differences with respect to general self-efficacy (measured with the GSE; neutral: *M* = 29.91, SD = 3.68, negative: *M* = 30.85, SD = 4.37, *p* = 0.35) and the ERQ subscales reappraisal (neutral: *M* = 4.89, SD = 1.13, negative: *M* = 4.81, SD = 1,03, *p* = 0.76) and suppression (neutral: *M* = 3.3, SD = 1.16, negative: *M* = 3.55, SD = 1.34, *p* = 0.42).

In addition, the intensity of the US (neutral: *M* = 4.23, SD = 1.49, negative: *M* = 4.27, SD = 1.37, *p* = 0.93) and its perceived aversiveness (US valence; neutral: *M* = 70.82, SD = 16.0, negative: *M* = 65.97, SD = 21.3, *p* = 0.3) was not subjected to group differences.

### Manipulation Check

Descriptive and test statistics of the VAS scales are shown in Table 1. The expected time × group interaction on the perceived coping capability scale of the VAS attained statistical significance. Analysis of simple effects showed that this interaction was driven by a significant decrease from pre- to post-induction in the negative (*p* < 0.001) but not in the neutral condition (*p* = 0.74). No other item on the VAS was subjected to group differences (i.e., main effect for group and time × group interaction, all *p* > 0.05; cf. Table 1).

**Table 1:** Descriptive and test statistics for the five VAS scales.

	Neutral		Negative				Time		Group		Time × group	
	*M*	SD	*M*	SD	*T*	*p*	*F*	*p*	*F*	*P*	*F*	*p*
**Distraction**							3.63	0.06	0.2	0.9	3.58	0.06
Pre	24.91	16.65	21.36	19.77	0.79	0.43						
Post	24.94	18.72	29.55	21.47	−0.93	0.36						
**Excitement**							1.24	0.27	0.02	0.89	2.9	0.09
Pre	34.85	24.96	31.18	24.59	0.60	0.55						
Post	33.30	25.80	38.55	23.58	−0.86	0.39						
**Positive mood**							5.32	0.02	0.07	0.79	0.16	0.69
Pre	72.39	16.09	74.09	15.58	−0.44	0.66						
Post	69.09	19.67	69.42	16.78	−0.07	0.94						
**Negative mood**							0.63	0.43	0.13	0.72	0.84	0.36
Pre	17.82	18.18	18.24	19.40	−0.09	0.93						
Post	21.24	19.28	18.00	15.06	0.76	0.45						
**Perceived coping capability**							10.4	0.002	0.19	0.66	7.58	0.008
Pre	66.61	16.53	72.52	18.35	−1.37	0.17						
Post	65.91	17.40	63.70	19.97	0.48	0.63						

### Fear Conditioning

#### CS-US Contingency Ratings

**Figure 2:**
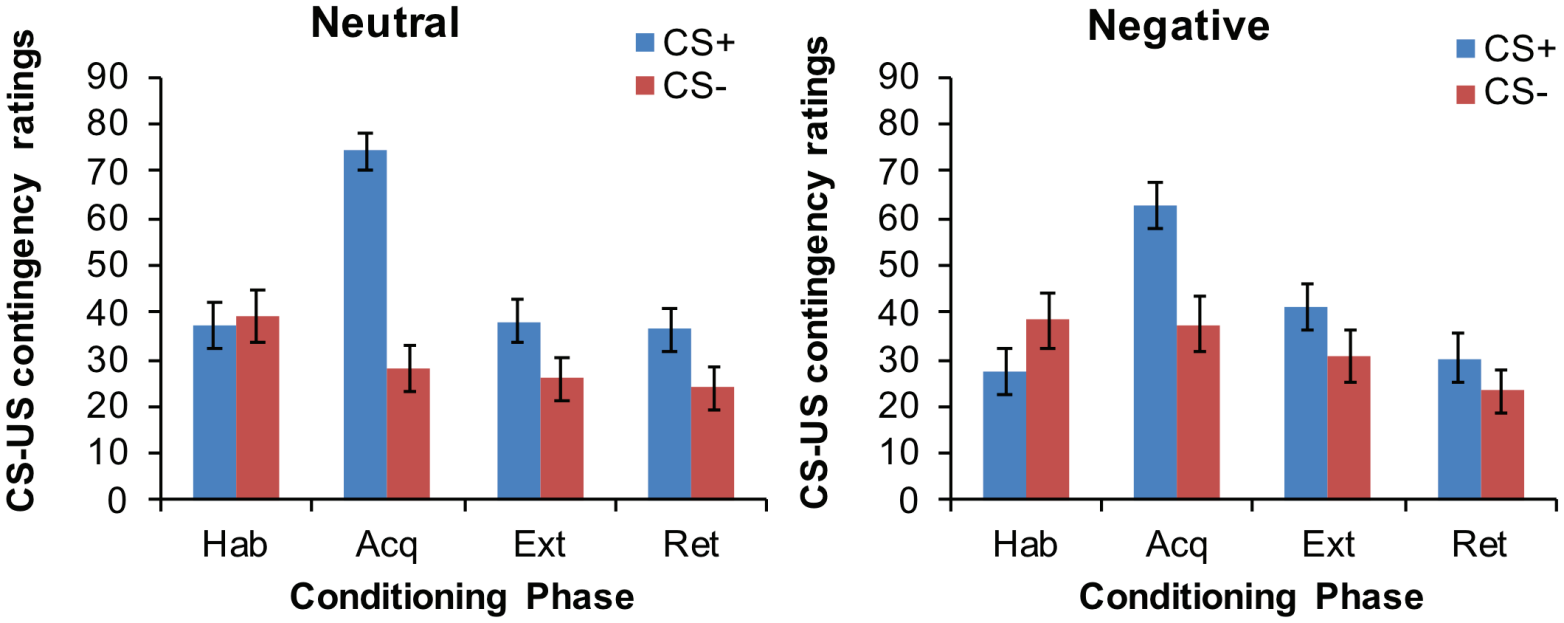
Mean CS-US contingency ratings at the end of each conditioning phase in the neutral and negative group.

After habituation, participants rated the two CSs differently, with higher ratings for the CS− relative to the CS+ (main effect for CS: *F*_(1,64)_ = 4.03, *p* = 0.049). After acquisition, the CS+ was rated as being more likely to be paired with the US than the CS− (main effect for CS: *F*_(1,64)_ = 63.95, *p* < 0.001), indicating successful acquisition. Interestingly, this CS+/CS− differentiation was subjected to group differences (CS × group interaction: *F*_(1,64)_ = 5.67, *p* = 0.02), with the negative group showing less discrimination among the CSs than the neutral group (cf. Figure 2). However, groups were still comparable in their absolute ratings attributed to the CS+ (*p* = 0.067) and CS− (*p* = 0.204). Participants continued to attribute higher ratings to the CS+ compared to the CS− after both extinction (main effect CS: *F*_(1,64)_ = 12.7, *p* = 0.001) and retrieval (*F*_(1,64)_ = 12.05, *p* = 0.001), with no group differences (main effect for group or group × CS interaction, all *p* > 0.32).

#### CS Valence

**Figure 3:**
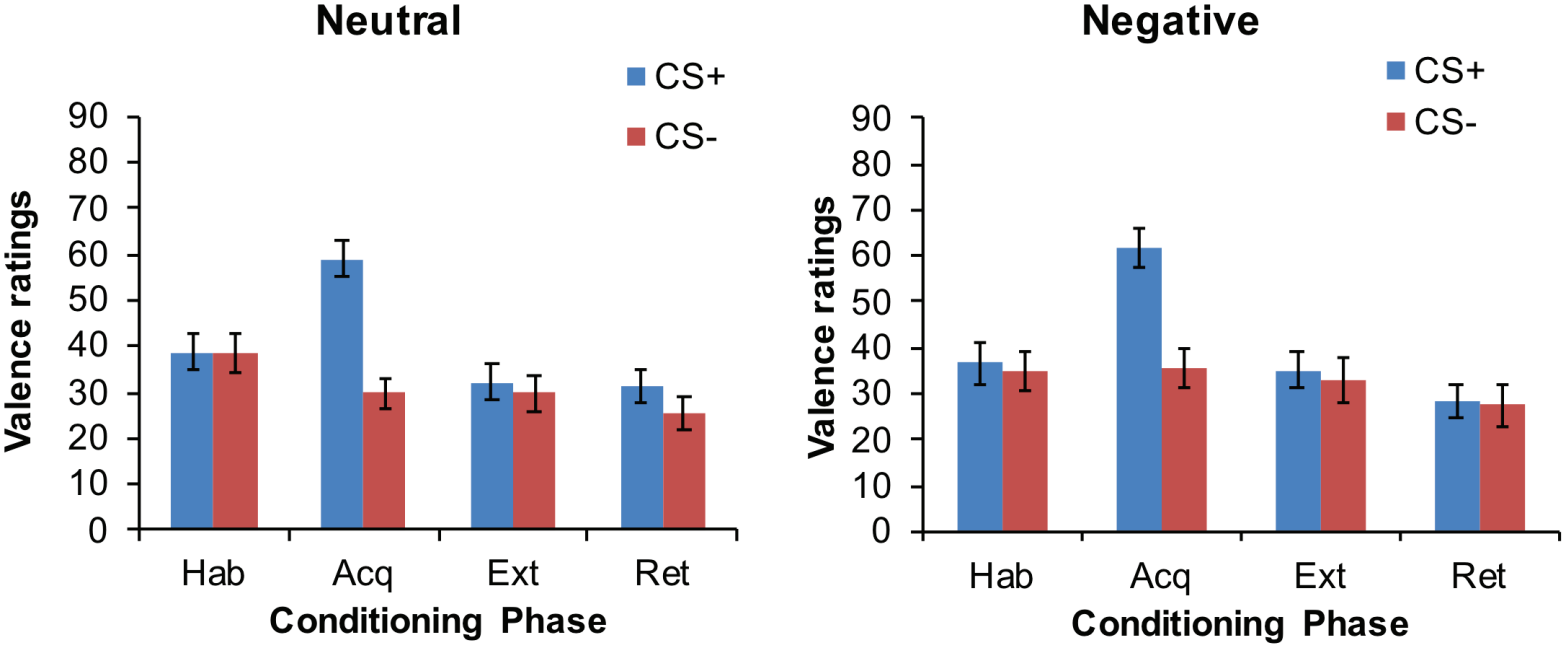
Mean CS valence ratings at the end of each conditioning phase in the neutral and negative group.

Data are displayed in Figure 3. Participants did not rate the CS+ and CS− differently after habituation. The CS+ was rated as more unpleasant than the CS− after acquisition (main effect for CS: *F*_(1,64)_ = 47.78, *p* < 0.001). This CS differentiation was no longer evident after extinction and retrieval, all *p > 0.*05. Main effects for group and time × group interactions did not attain statistical significance either, all *p* > 0.05.

#### SCR

**Figure 4:**
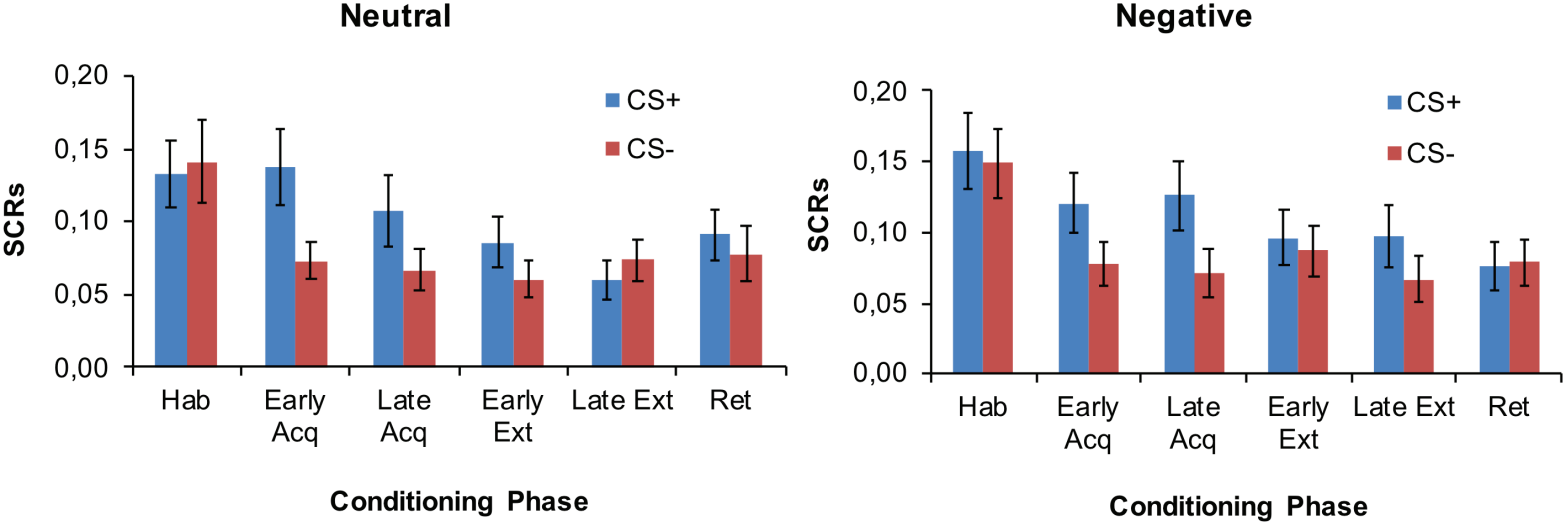
Mean SCRs during each phase of conditioning in the neutral and negative group.

Due to equipment malfunction, SCR data from *n* = 2 participants were lost and SCR data from *n* = 3 and *n* = 2 subjects were not recorded during habituation and during both habituation and acquisition, respectively. Hence, the analytic sample comprised *N* = 59 participants during habituation, *N* = 62 during acquisition, and *N* = 64 during extinction and retrieval. Data are depicted in Figure 4. During habituation, SCRs to the CS+ and CS− were comparable, with no group differences. Fear acquisition was successful as indicated by a significant main effect for CS (*F*_(1,60)_ = 23.31, *p* < 0.001). No effects for block, group or any interaction were significant during acquisition (all *p* > 0.25). The differentiation between the CS+ and CS− was no longer evident during extinction or during retrieval and no other main effects or interaction effects were observed (all *p* > 0.12).

## Discussion

The present study sought to investigate the effect of an experimentally induced low perceived self-efficacy on associative learning in a differential fear conditioning task. Our results suggest that the negative verbal feedback was effective in the way that it led to a decreased perceived self-efficacy relative to the neutral feedback. Additionally, there was a tendency toward an increased distraction as well as an increased excitement in the group receiving the negative feedback although the group × time interaction was not significant. Most importantly, the group receiving the negative feedback showed changes in the acquisition of conditioned fear responses. The pattern of findings suggests a selective effect of the negative self-efficacy manipulation on discriminative learning during fear acquisition. In particular, a lower self-efficacy in the group receiving a negative feedback seems to alter the ability to learn the contingency between the different CSs, i.e., to estimate the probability of receiving a shock following the presentation of the CS+ versus the CS−.

While diminished fear discrimination learning has been demonstrated in anxious individuals previously, this was usually due to an increased responding to the safety cue (CS−) during fear acquisition (Hermann et al., 2002; Lissek et al., 2009). The present findings, however, rather suggests that the diminished discrimination in the low self-efficacy group is due to less discrimination between the CS+ and CS− during fear acquisition. This response pattern is comparable to findings from studies investigating the impact of increased state anxiety on discrimination learning. Recently, Dibbets and Evers (2017) demonstrated that higher state anxiety levels (induced by the Trier Social Stress Test task) lead to less discrimination between the CS+ and CS− for the US expectancies during fear acquisition. Similarly, Vriends et al. (2011) reported a decreased discriminative learning during fear acquisition in individuals with an experimentally induced increased state anxiety. Thus, one might suggest that the group receiving the negative self-efficacy feedback exhibits increased state-anxiety which, in turn, decreased discrimination learning. In line with this hypothesis, a systematic manipulation of self-efficacy levels *via* verbal feedback has indeed been shown to affect anxiety and arousal levels (Marquez et al., 2002). Interestingly, the effect of low self-efficacy on fear conditioning was displayed on the level of CS-US contingency measures. This again is partially in line with findings from studies showing an effect of increased state anxiety on discrimination between CS+ and CS− on measures of US expectancy during fear acquisition, but no effects on the level of electrodermal responding (SCRs) (Dibbets and Evers, 2017). While these findings support the hypothesis of a common mechanism underlying the effects of increased state anxiety and low self-efficacy on discrimination learning, our negative feedback intervention did not lead to changes in negative mood. This suggests that the effects of low self-efficacy on discriminative fear learning might not be solely influenced by changes in state anxiety or mood. Our results point to a slightly different pattern of findings relative to studies which examined the impact of increased state anxiety on fear conditioning (Vriends et al., 2011; Dibbets and Evers, 2017). The current study revealed no effects of the self-efficacy manipulation on negative mood. Ratings on negative mood and state anxiety, however, do not necessarily overlap. Given the close link between self-efficacy and state anxiety (Marquez et al., 2002) the inclusion of measures of state anxiety in future studies on self-efficacy manipulations would be desirable.

Nevertheless, the absence of effects on other outcome measures is interesting and suggests that a lower self-efficacy alters cognitive aspects of discrimination (but not affective components and physiological responding) during fear learning. The discordance or desynchrony in fear measures generally supports the propositions of the dual-process model of fear learning. Briefly, different mechanisms contribute to affective and expectancy learning (Baeyens et al., 1995; Hamm and Vaitl, 1996; Hamm and Weike, 2005). These two types of learning processes should be reflected by different cognitive processes and mediated by different neuronal structures. For instance, expectancy learning requires individuals to detect ambiguous cues during fear learning and associate these cues with different outcomes. Systematic investigations using different conditioning procedures suggest that these (conscious) cognitive processes require intact hippocampal and prefrontal structures (Clark and Squire, 1998; Connor and Gould, 2016). In contrast, automatic and implicit lower level processes are required for assigning the affective meaning of the US valence to the different CSs during fear learning. Thus, according to the dual process models, a lower self-efficacy might be associated with an impaired ability to estimate and assign different outcomes to the safety and danger cues during fear learning. It should be noted that the expectancy ratings in our study were collected after the completion of the acquisition phase. Concomitant analysis of electrodermal data during the fear acquisition phase per se suggests that participants receiving the negative feedback dissociated the CS+ and CS−. One might therefore wonder why a lower self-efficacy impairs the ability to estimate the probability to receive shocks upon presentation of the different CSs after fear acquisition.

Perceived self-efficacy in our study was operationalized by providing a discrete feedback to participants regarding their general ability to cope in demanding situations (see Brown et al., 2012a,b). Thus, in line with the central idea of the self-efficacy theory, participants in the low self-efficacy group are characterized by a decreased perceived belief that they can exert adaptive behavior or that their behavior during stressful conditions will less likely produce a positive outcome. In order to be able to show adaptive behaviors, it is important to estimate the different outcomes in response to discrete stimuli from the environment. The ability to monitor discrete cues from the environment and establish stimuli-outcome associations is dependent on intact executive functions including working memory capability (Carter et al., 2003). Acute stress and distraction can alter working memory processes which results in a detrimental effect on associative learning (Carter et al., 2003; Raio and Phelps, 2015). For instance, prior experiences with unpredictable/uncontrollable stressors have been shown to impair discriminative fear learning (Meulders et al., 2012). Meulders et al. (2012) showed that unpredictability experiences affect later fear conditioning by blocking discriminative fear learning. Moreover, the effects of prior exposure to unpredictable stressors was expressed by a lack of differential CS-US shock-expectancy ratings, but not on the level of psychophysiological responding (fear-potentiated startle (Meulders et al., 2012), which is similar to the herein observed divergence in fear measures after the negative feedback intervention. Thus, one might speculate that both prior unpredictable stress and low self-efficacy lead to a state of increased distraction and uncertainty which results in a diminished ability to establish different CS-US outcomes. In line with this assumption, our analyses revealed an increased distraction in the group receiving the negative feedback (cf. Table 1), which might have mediated the effect of low self-efficacy on discriminative fear learning.

Future studies, however, are needed to delineate the link between low self-efficacy, working memory, and discriminative fear learning. It also remains unclear why low-self-efficacy alters the process of fear acquisition but not fear extinction and/or extinction retrieval. One obvious explanation might be that the selective effects of low self-efficacy on fear acquisition are due to the distinct cognitive and neurobiological mechanisms underlying fear acquisition and fear extinction. In this instance, it is notable that the present study builds on our previous work assessing the impact of increased self-efficacy on fear conditioning (Zlomuzica et al., 2015). However, a direct comparison to Zlomuzica et al. is not possible for several reasons. First, in Zlomuzica et al., the impact of increased self-efficacy on fear extinction, but not fear acquisition was examined. Second, the experimental design in Zlomuzica et al. (2015) did not include a comparison group who received a neutral feedback. The present study included a direct comparison group which received a neutral verbal feedback. Providing a neutral verbal feedback (relative to a study which does not include a neutral verbal feedback, see Zlomuzica et al., 2015) can influence group differences in mood, arousal, and self-efficacy ratings. On the contrary, distraction does not seem to be affected by a positive self-efficacy intervention (see Zlomuzica et al., 2015). Nevertheless, the present findings and those by Zlomuzica et al. demonstrate in a similar way that changes in self-efficacy can indeed alter the processes of fear acquisition and fear extinction, which has a high clinical value for further research on the development and treatment of pathological fear and anxiety. Low self-efficacy is systematically related to an increased fear and avoidance behavior in anxiety disorders (Williams and Watson, 1985). Accordingly, a decrease in phobic behavior goes along with an increase in perceived belief to cope with threatening situations (Williams et al., 1985). Cognitive behavioral therapy is associated with an enhanced self-efficacy belief which might explain reductions in psychopathological symptoms following successful treatment (Bouchard et al., 2007; Gaudiano and Herbert, 2007; Delsignore et al., 2008). Thus, one might suggest that a decreased self-efficacy hampers the beneficial effects of cognitive behavioral treatment on fear and avoidance in various anxiety and stress-related disorders (Bouchard et al., 2007; Goldin et al., 2012; Gallagher et al., 2013).

Our findings might also contribute to a better understanding on the impact of cognitive processing during associative learning. For instance, a number of studies that predominantly used verbal instructions prior to fear acquisition and extinction in Pavlovian conditioning (Mertens et al., 2018a) demonstrated distinct and dissociable effects of verbal instructions on affective and expectancy learning. The herein presented results reveal new important insight into the link between cognitive processing during states of low self-efficacy and modulation of CS-US contingency learning (Boddez et al., 2013; Mertens et al., 2018b). Interestingly, verbal instructions can lead to an increased discrimination of fear responses (Duits et al., 2017). Increased ability to discriminate between danger and safety cues might predict better exposure-based treatment efficacy which requires an intact ability to establish stimulus-threat associations (Duits et al., 2017). Together with these prior findings, our study points to a new, yet largely unexplored effect of self-efficacy belief on discriminative fear learning. The instructions regarding the CS-US-contingencies during the fear conditioning task were chosen according to existing protocols from the own lab (Mosig et al., 2014; Zlomuzica et al., 2015). In the present study, an instruction that did not include explicit information regarding the direction of CS-US contingency (i.e., which of the two CSs will be paired with the UCS and which CS will never be paired) was provided. This procedure represents one possibility how to present instructions on CS-US contingency prior to fear learning. However, there are considerable variations of this procedure, and the type of instructions regarding the relationship between the CS and US can have a profound impact on fear learning and fear expression (see Lonsdorf et al., 2017).

The study has a number of limitations. First, although the construct of self-efficacy has received great interest in clinical research, the effects of a short negative verbal instruction and a chronic state of low self-efficacy (e.g., a history of repeated, negative verbal feedbacks and/or the absence of mastery experiences) is not comparable. Thus, the extrapolation of findings from our experimental manipulation to a more clinically relevant low self-efficacy is difficult. Second, the transfer of findings in our task to similar learning experiences in real-life situations is also lacking. Third, since we tested healthy subjects with an experimentally induced level of low self-efficacy, it is not clear whether a similar effect in clinical samples characterized by low self-efficacy levels could be observed. Finally, it remains unclear why our intervention did not affect extinction learning. Since the experimental manipulation was introduced prior to the fear acquisition, one explanation is that the effects of the self-efficacy intervention might not be long-lasting. A more elaborated experimental design including a group receiving the negative feedback prior to fear extinction (i.e., after fear acquisition) would be needed together with repeated measurements of negative/positive mood, distraction, excitement, and perceived self-efficacy during the distinct phases of fear acquisition, extinction, and extinction retrieval.

To conclude, verbal persuasion might have only a small effect on the level of perceived self-efficacy compared to mastery experiences or other sources of self-efficacy (Bandura, 1977). Therefore, manipulation of self-efficacy *via* different sources might be interesting to examine its role on associative learning. In this instance, we (Raeder et al., 2019) and others (Morina et al., 2017) have recently investigated the possibility to increase self-efficacy trough reactivation of personal mastery experiences. Future studies incorporating such manipulations of self-efficacy during fear conditioning would be interesting to extend our understanding on the functional link between self-efficacy and associative learning. Likewise, in order to derive meaningful conclusions on the effect of low self-efficacy on psychopathology, specifically designed studies in clinical population would be needed. It is reasonable to assume that low perceived self-efficacy in clinical populations can be hardly mimicked by any experimental manipulation. Decreased self-efficacy in individuals with clinically relevant fear and anxiety might be functionally linked to the observed symptomatology (Williams and Watson, 1985; Jones and Menzies, 2000). Individuals with clinical relevant anxiety show alterations in fear learning and extinction (Lissek et al., 2005). Interestingly, there is some evidence that individuals with anxiety disorders show an altered ability to estimate aversive outcome following both fear-relevant and fear-irrelevant stimuli (Duits et al., 2016). It would be interesting to examine the contribution of perceived self-efficacy to such threat expectancy bias in clinical population. Furthermore, since techniques to increase self-efficacy in clinical samples (Morina et al., 2017; Raeder et al., 2019) have been developed, one might examine how these manipulations affect the ability to estimate the probability of stimulus-threat associations in clinical anxiety.

Our study provides experimental evidence that low self-efficacy is associated with a diminished discriminative fear learning. Low efficacy alters outcome of stimuli signaling danger and safety. Such alterations might hamper the subject’s ability to estimate positive and negative outcomes related to discrete stimuli from the environment and adaptively interact with threatening and demanding situations.

## Ethics Statement

This study was carried out in accordance with the recommendations of the Ethics Committee of the Ruhr University Bochum. All subjects gave written informed consent in accordance with the Declaration of Helsinki. The protocol was approved by the local ethics committee of the Ruhr University Bochum.

## Author Contributions

FR and AZ designed the research, and FR, LK, and HS performed the research. FR and AZ analyzed the data. FR and AZ wrote the manuscript. FR, LK, HS, JM, and AZ provided critical comments and approved the manuscript.

### Conflict of Interest Statement

The authors declare that the research was conducted in the absence of any commercial or financial relationships that could be construed as a potential conflict of interest.
